# Dapsone-Induced Hypoxia

**DOI:** 10.7759/cureus.9334

**Published:** 2020-07-22

**Authors:** Dinesh Keerty, Kevin Eaton, Elizabeth Haynes

**Affiliations:** 1 Internal and Hospital Medicine, H. Lee Moffitt Cancer Center and Research Institute, Tampa, USA; 2 Internal Medicine, Moffitt Cancer Center, Tampa, USA; 3 Internal and Hospital Medicine, Moffitt Cancer Center, Tampa, USA

**Keywords:** drug-related side effects and adverse reactions, methemoglobinemia, dapsone, hypoxia

## Abstract

Dapsone is a common medication that is utilized in the treatment of dermatological conditions, pneumocystis pneumonia, and toxoplasmosis. Methemoglobinemia is a known but rare complication of dapsone therapy that can result in cyanosis. We present a case of a patient on dapsone therapy who developed hypoxia due to methemoglobinemia. This case emphasizes the importance of knowledge of drugs likely to cause methemoglobinemia which requires clinicians to have a high degree of suspicion especially when the patient's oxygen saturation does not improve with treatment.

## Introduction

Since the 1940s, the World Health Organization has approved the usage of dapsone as the principal drug in a multi-drug regimen for the treatment of leprosy [1-2]. Dapsone is also utilized in the treatment of numerous dermatological conditions, pneumocystis pneumonia, and toxoplasmosis [3-4]. Dapsone has been noted as uniquely effective against dermatitis herpetiformis, with such a significant response to pruritus that it was even considered as a diagnostic indicator [5]. Dapsone causes numerous adverse effects involving all organ systems in various patient populations. Some of the noted side effects are agranulocytosis, nephritis, hypoalbuminemia, hypersensitivity syndrome, Steven-Johnson syndrome, and methemoglobinemia [6]. Methemoglobinemia is a known but rare complication of dapsone therapy that can result in cyanosis [1, 3-4]. It is a potentially fatal condition when diagnosis is not made in a timely manner. We are here to present a case of a patient on dapsone therapy who developed hypoxia due to elevated methemoglobin levels.

## Case presentation

**Figure 1:**
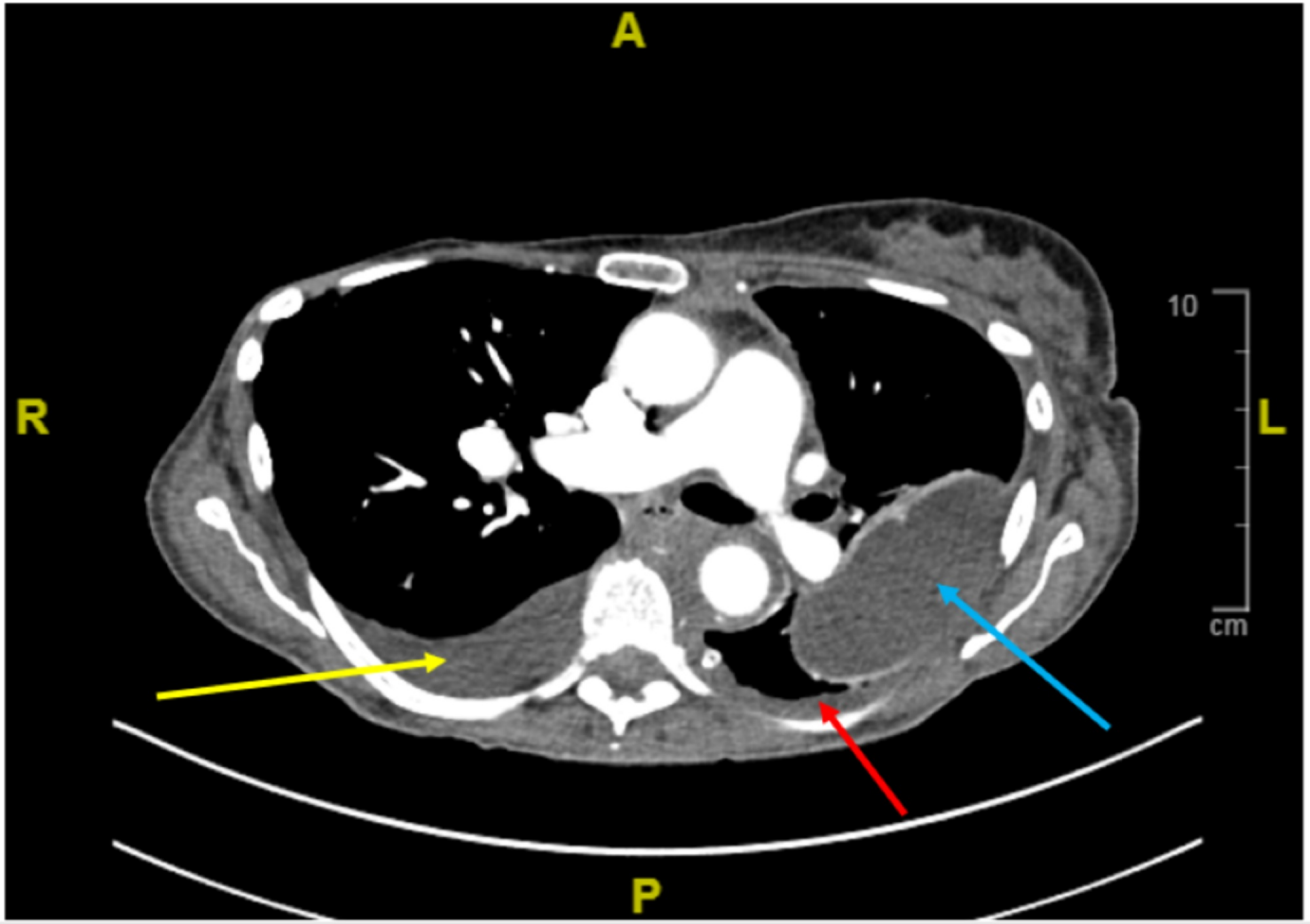
CT pulmonary angiogram. Pulmonary angiogram cross-section at level of pulmonary artery bifurcation. Yellow arrow: There is a moderate right pleural effusion which is increased in extent from prior exam. Blue arrow: There has been an interval decrease in size of the loculated left pleural effusion. Red arrow: There is left-sided pleural thickening and asymmetric soft tissue along the left hemi-diaphragm which demonstrated increased fluorodeoxyglucose (FDG) uptake on recent positron emission tomography (PET) scan examination.

A 76-year-old female with acute myeloid leukemia (AML) presented to the ED with complaints of shortness of breath on exertion. Her past medical history is significant for breast cancer for which she had surgery and adjuvant chemotherapy 10 years ago. She was diagnosed with mixed phenotype AML about two years ago and has been treated with cyclophosphamide, methotrexate, doxorubicin, vincristine, and cytarabine for three cycles. As there was no morphologic evidence of leukemia, she was then placed on maintenance therapy with 6-mercaptopurine, vincristine, methotrexate, and prednisone (POMP) for one year. During the past year, she developed febrile neutropenia with pneumonia and sepsis which required her to discontinue POMP in the last three months. After a three-month hiatus in treatment, she was noted to have recurrent pleural effusions which showed relapsed mixed phenotype acute leukemia. She was admitted for chemotherapy initiation with intra-thecal methotrexate and blinatumomab two weeks prior to the ER presentation. During her admission, she had placement of pleural catheter for recurrent left-sided pleural effusions. She tolerated the procedure and concurrent chemotherapy relatively well. She was discharged home to continue outpatient treatment. Prior to discharge she was started on dapsone as part of her anti-microbial prophylaxis, as she developed renal insufficiency and hyperkalemia with sulfamethoxazole-trimethoprim. A week after discharge, she presented with acute shortness of breath aggravated with minimal exertion. Her initial oxygen saturation was 85% on room air prompting immediate placement of 2 L of oxygen via nasal cannula. Her oxygen saturation improved only to 90%, and further increase in oxygenation did not show improvement in saturation. She had drained 100 mL of fluid from her pleural catheter one day prior. Our initial suspicion was she developed either an acute pulmonary embolism or loculated pleural effusion. We performed a pulmonary angiogram which showed no pulmonary embolus, but a new moderate pleural effusion on the right side and left-sided pleural thickening with minimal fluid in the pleural space (Figure 1).

**Figure 2:**
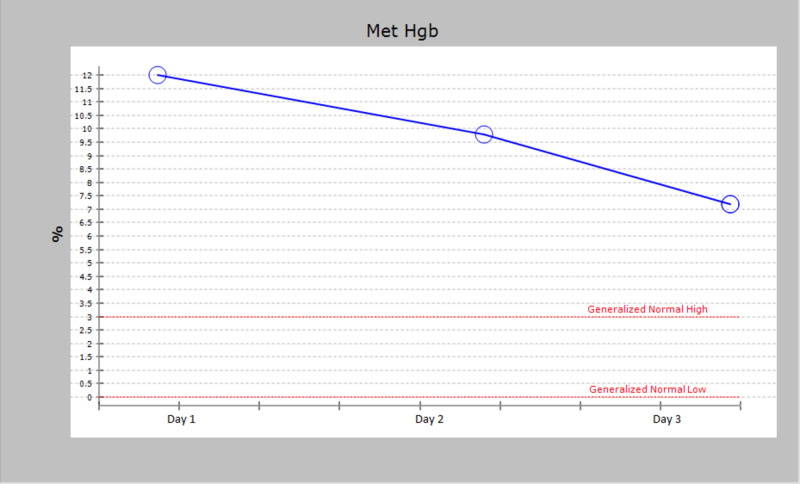
Methemoglobin % in arterial blood gas analysis. Down trending methemoglobin % noted over a span of three days

Thoracentesis was performed on the right pleural space with removal of 300 mL of fluid. The patient’s oxygenation saturation did not improve at all. We obtained an arterial blood gas to evaluate her oxygenation status and it showed pH of 7.49, PCO2 30, PaO2 133, methemoglobin of 12%. As the patient was noted to be on dapsone, the correlation of dapsone-induced methemoglobinemia causing hypoxia was made. Upon further re-examination, we noted slight grayish-blue discoloration of her fingers. We switched the dapsone with atovaquone and admitted her to monitor methemoglobin levels. Serial arterial blood gases showed significant improvement of her methemoglobin levels over the span of three days (Figure 2).

The patient did not require treatment with methylene blue as she was not in acute distress. Her oxygen saturation improved to 94% on room air by the third day. She was subsequently discharged home to resume her chemotherapy. She has been tolerating her atovaquone therapy well and has not had similar complaints.

## Discussion

Dapsone is absorbed rapidly from the gastrointestinal tract with peak plasma concentrations within two to eight hours after administration. The half-life of elimination is about 20-30 hours [7-8]. Hemoglobin transports oxygen in blood. Hemoglobin when stressed due to hereditary defects or oxidative processes leads to formation of methemoglobin. Normally, our body reduces this methemoglobin to hemoglobin via cytochrome b5 reductase. However, the balance can be tipped when exposure occurs to various oxidizing agents such as benzocaine, lidocaine, dapsone, choloroquine, and other dyes [9-10].

Acquired methemoglobinemia from dapsone therapy results in an inability of hemoglobin to bind oxygen causing reduced oxygen delivery [3]. Prolonged reduction in oxygen delivery results in diffuse tissue hypoxia and cell death. Normal human red blood cells have methemoglobin levels of less than 2% [4]. Elevation in methemoglobin levels are noted in congenital enzyme deficiencies in patients with cytochrome b5 reductase deficiency and patients with hemoglobin M [11]. Clinical signs and symptoms of methemoglobinemia vary by percentage in blood concentration [4, 12] (Table 1).

**Table 1:** Common symptoms of methemoglobin based on arterial blood gas percentage.

Methemoglobin level (%)	Common symptoms
< 3	None, normal physiology
3-10	Asymptomatic
10-20	Symptomatic, peripheral and central cyanosis
21-50	Headache, fatigue, tachycardia, weakness, dizziness
>50	Cardiac arrhythmia, dyspnea, seizures, coma, death

Diagnosis of methemoglobinemia requires a high degree of clinical suspicion. Peripheral pulse oximetry does not detect methemoglobin and can reflect falsely elevated or decreased oxygen saturation levels. The most definitive evaluation is by the use of arterial blood gas analysis. Methylene blue is the suggested antidote which is utilized in patients with methemoglobin percentage greater than 30% or severely symptomatic. It works by reducing methemoglobin to hemoglobin. In case with concurrent G6PD deficiency, methylene blue is contraindicated and instead hyperbaric oxygen or exchange transfusions are recommended alternatives [4].

## Conclusions

This case emphasizes the importance of knowledge of drugs likely to cause methemoglobinemia that would require clinicians to have a high degree of suspicion, especially when the patient's oxygen saturation does not improve with oxygen. Prompt evaluation with arterial blood gas analysis is essential for the best outcomes. 
